# Network-based features enable prediction of essential genes across diverse organisms

**DOI:** 10.1371/journal.pone.0208722

**Published:** 2018-12-13

**Authors:** Karthik Azhagesan, Balaraman Ravindran, Karthik Raman

**Affiliations:** 1 Department of Biotechnology, Bhupat and Jyoti Mehta School of Biosciences, Indian Institute of Technology (IIT) Madras, Chennai – 600 036, India; 2 Department of Computer Science and Engineering, IIT Madras, Chennai – 600 036, India; 3 Initiative for Biological Systems Engineering (IBSE), IIT Madras, Chennai – 600 036, India; 4 Robert Bosch Centre for Data Science and Artificial Intelligence (RBCDSAI), IIT Madras, Chennai – 600 036, India; National Centre For Cell Science, INDIA

## Abstract

Machine learning approaches to predict essential genes have gained a lot of traction in recent years. These approaches predominantly make use of sequence and network-based features to predict essential genes. However, the scope of network-based features used by the existing approaches is very narrow. Further, many of these studies focus on predicting essential genes within the same organism, which cannot be readily used to predict essential genes *across* organisms. Therefore, there is clearly a need for a method that is able to predict essential genes across organisms, by leveraging network-based features. In this study, we extract several sets of network-based features from protein–protein association networks available from the STRING database. Our network features include some common measures of centrality, and also some novel recursive measures recently proposed in social network literature. We extract hundreds of network-based features from networks of 27 diverse organisms to predict the essentiality of 87000+ genes. Our results show that network-based features are statistically significantly better at classifying essential genes across diverse bacterial species, compared to the current state-of-the-art methods, which use mostly sequence and a few ‘conventional’ network-based features. Our diverse set of network properties gave an AUROC of 0.847 and a precision of 0.320 across 27 organisms. When we augmented the complete set of network features with sequence-derived features, we achieved an improved AUROC of 0.857 and a precision of 0.335. We also constructed a reduced set of 100 sequence and network features, which gave a comparable performance. Further, we show that our features are useful for predicting essential genes in new organisms by using *leave-one-species-out* validation. Our network features capture the local, global and neighbourhood properties of the network and are hence effective for prediction of essential genes across diverse organisms, even in the absence of other complex biological knowledge. Our approach can be readily exploited to predict essentiality for organisms in interactome databases such as the STRING, where both network and sequence are readily available. All codes are available at https://github.com/RamanLab/nbfpeg.

## Introduction

Proteins perform a plethora of different functions in every living cell. It is interesting to understand the role of different proteins and their importance, in terms of their essentiality for a cell to survive. Several proteins orchestrate critical cellular functions. These proteins are consequently indispensable for the survival of a cell; the genes encoding these proteins are thus *essential genes*. Essential genes have also been defined as those genes indispensable for reproductive success, either at a cellular or organismal level [1]. The applications of essentiality are varied, ranging from finding the minimal genome required for sustenance to ranking drug targets [1, 2]. Essential genes have been experimentally identified using transposon mutagenesis, anti-sense RNA, RNA interference and single gene deletion [2]. However, experimental determination of essential genes is expensive, time-consuming and laborious [3]. Computational predictions of essential genes can give a prioritised shortlist for experimental validation.

Essentiality of a gene is conditionally dependent on various factors such as growth conditions, medium, developmental stage, and genetic context [1, 3]. Gene essentiality is challenging to predict since it encompasses a number of factors. Nevertheless, many studies have sought to predict essentiality using machine learning approaches. For this purpose, essentiality was defined as indispensability of a gene under rich media conditions [4]. Predicting essential genes and uncovering novel aspects responsible for essentiality could fundamentally improve our understanding of an organism’s behaviour. Experimentally, data on essential genes are available only for a few organisms. On the other hand, sequence and protein–protein interaction (PPI) network information are available for several organisms, making it feasible for large-scale *in silico* predictions.

Different *in silico* approaches have been developed to predict essential genes [4]. In particular, machine learning approaches have been developed to predict essential genes across and within organisms, using sequence, network and metabolic features [3]. Some examples of sequence-based features that are found to be useful for predicting essential genes are ZCURVE features from DNA sequence [5], 60 physicochemical and other sequence-based features from protein and DNA sequence [6], fractal features [7] and information-theoretic features [8]. Hwang *et al*. combined different network and sequence-based features to predict essential genes [9]. Further, many approaches have combined sequence, network and biological information [10, 11] and identified properties such as Domain Enrichment Score (DES), which is computed based on the fraction of essential domains, to be highly useful [10]. Other approaches have modified naïve Bayes classifier [12], and developed new algorithms to predict essential genes using features such as strand bias, homology and Codon Adaptation Index (CAI) [13]. A review of different computational methods tried so far to predict essential genes both within and across organisms using network features can be found in [3].

Uncovering the network aspects responsible for essentiality without using any complex biological information could help us unravel the significance of network structure and their importance in essentiality across various organisms. PPI networks are widely available, and databases such as STRING consist of known and predicted protein–protein associations data for more than 2000 organisms (version 10 [14]). In addition, the Database of Essential Genes (DEG) has essential genes data for over 50 organisms (version 15.2 [15]), and Online GEne Essentiality (OGEE) database has essentiality data for 48 organisms (as of February 2018) [16]. Given the availability of data on essential genes, as well as network information from databases such as the STRING, there exists a need to develop effective methods for classification of essential genes, that make use of network-based features. Extracting features based on network information and using them to predict gene essentiality can enable bridging the gap between organisms with known essential genes and interactome information.

Previous essentiality studies have not focused enough on aspects of the network organisation, and the role that ‘network position’ plays in essentiality. On the other hand, in the field of social networks, recent studies have illustrated the importance of network position and network properties in determining the structural roles played by different nodes in the network [17–19]. Regional features, as computed by ReFeX [17], recursively capture the properties of a neighbour and a neighbour’s neighbour and so on. In the present study, we adapted these ideas to predict essential genes across diverse organisms. Moreover, there is no extensive study so far that has focused on analysing different network-based properties across protein interaction networks of diverse organisms, for predicting essential genes.

In this context, we propose to identify robust network-based features that can capture network structure for better prediction of essential genes across organisms. The key questions that we seek to address are: Are network-based features more effective than sequence-based features? Is it possible to predict essential genes across PPI networks of different organisms, which differ widely in the numbers of nodes and edges, as well as numbers of essential and non–essential genes? Can the essentiality of proteins be predicted predominantly by their position in the network? Does augmenting sequence-based to network-based features yield any improvement in the overall performance of essential genes prediction? To address these questions, we studied PPI networks of 27 diverse bacterial species. We show that our proposed network features are effective in predicting essential genes across diverse organisms and are better than sequence and other conventional network features, such as degree centrality and clustering coefficient. Notably, we show a significant increase in performance over the features used in existing methods such as ZUPLS [5] and the features proposed by Liu *et al*. [6]. We also show that augmenting sequence-based features with network-based features yields further improvement in performance. Further, we show that our features are effective using *leave-one-species-out* validation.

## Methods

In this section, we first outline the datasets used in this study and then describe in detail the network-based features that we have employed, to predict essential genes across organisms.

### Datasets

Data on essential genes are available from the DEG database for over 50 organisms (version 15.2, as of February 2018) [15]. However, to enable a systematic comparison with the recent state-of-the-art, such as the work by Liu *et al*. [6], we restrict our studies to 31 prokaryotic organisms from DEG version 11.1. Out of 31 organisms, only 27 organisms had PPI data available in the STRING database (version 9.1 [20]). Further, out of 103,624 genes across these 31 organisms, only 87,159 (84%) had known interactions in STRING. A list of all 27 organisms, along with the statistics on the number of nodes and edges in each network is available in S1 Table.

### Recursive feature extraction (ReFeX)

ReFeX is a recursive feature extraction technique that has been previously shown to enable transfer of class labels across networks from various domains [17]. Each PPI network that we take into consideration has a certain structure. We hypothesise that essential genes across networks, i.e. across organisms, share “structural”/network features that are effective in transferring essential gene labels across networks. That is, these features capture the network structure of different organisms and may hence be useful in identifying essential genes in one organism, based on features/patterns learnt in another.

Since ReFeX has previously been shown to be useful in transferring labels across networks with a different number of nodes and edges, we employ ReFeX on our organism PPI networks. Overall, we compute 267 different ReFeX features that fall under three different categories: (i) *local features*, such as degree, which are local to a node, (ii) *egonet features*, which refer to the node and the induced subgraph formed by a node and all of its neighbours, and (iii) *regional features*, which are a recursive combination of local and egonet features. Recursive iterations of the means and sums of these local and egonet features are performed to capture the overall structural properties of the node. We used a total of 267 recursive features (for a detailed description, see S2 Table) to construct the “ReFeX feature set”.

### Network centrality measures

Many previous studies have explored the correlation between centrality and essentiality (or lethality) in biological networks [21–23]. The “centrality–lethality” hypothesis posits that nodes that are highly central in a network are much more likely to be lethal/essential [21]. In network analysis, centrality measures identify the central nodes based on certain parameters. Degree centrality, being the simplest of all the centrality measures, captures the number of immediate neighbours of a given node. Betweenness and load centralities compute the significance of a node by calculating the fraction of all shortest paths that pass through a node. Another set of centralities, eigenvector centrality and PageRank define the influence or the importance of a node in a network. Overall, we used the following “12 centrality measures” in our analyses: closeness centrality, betweenness centrality, degree centrality, eigenvector centrality, subgraph centrality, information centrality, random walk betweenness centrality, load centrality, harmonic centrality, reaching centrality, edge clustering coefficient centrality and PageRank. Detailed definitions of all these measures can be found elsewhere [24, 25]. We also combined clique number and clustering coefficient with the above-mentioned centrality measures and designated the set as “14 network measures”. We combined all the above network properties and used them as features for essential genes prediction. The final number of network features that we used are 283, ignoring the repeated properties: 267 ReFeX features, “12 centrality measures”, clique number, clustering coefficient, biconnected components and weighted degree (for a detailed listing of these features, see S3 Table).

### LASSO feature selection

We employed feature selection to identify key features from the list of 283 features mentioned above. For this, we employed the widely used LASSO (Least Absolute Shrinkage Selection Operator) technique [26]. LASSO employes an *L*_1_ regularisation to shrink the weights assigned to different features and make the set of weights sparse. This reduces the number of features with non-zero weights, and these features are subsequently selected for use in classification. We selected properties with non-zero weights for the best model using LASSO by doing 10-fold cross-validation on the entire set of 87,159 genes. This gave rise to 100 features, comprising ten centrality measures, clique number, clustering coefficient and 88 ReFeX features.

### Combined sequence and network properties

We also augmented the 283 network-based features described above with sequence-based features proposed in previous studies. Liu *et al*. evaluated 60 different features based on sequence and physicochemical properties and selected 40 features as useful for classification of essential genes. Another method, ZUPLS [5], used 274 different features based on sequence homology and other ZCURVE/sequence properties apart from the features described in Liu *et al*. A detailed description of these features is available in the original papers [5, 6]. This gives us a total of 597 features in all, viz. 283 network-based features, 40 top features from Liu *et al*. and 274 features from ZUPLS.

We again performed LASSO on this entire set of features and selected 300 features for further evaluation. The final selected features comprised 198 ZUPLS features, weighted degree, 38 features from Liu *et al*., seven centrality measures, clique number and 55 ReFeX features (for a detailed listing of these features, see S4 Table).

### *Leave-one-species-out* validation

We also evaluated our features using *leave-one-species-out* validation in which one species is left out as test set whereas all the other 26 species are kept as training set. We repeated this 27 times with different set of organisms as training and test set. This experiment was performed to check whether the features are effective to predict essential genes in a new unseen organism and are transferable across organisms. We used Random Forest Classifier [27] with 100 trees after undersampling equal number of non-essential genes since it was easily scalable for predicting essential genes in new organisms.

### Feature extraction

We extracted ReFeX and other network-based features for the 27 organism PPI networks that we obtained from the STRING database [20]. All the network properties were scaled using the “min-max scaler” from the SciKit Learn library for Python [28] since the networks are of different sizes. The scaled features were then used for classification. As discussed previously, we used 87159 genes that had available network information, for classification and comparison. We extracted ZUPLS features for the same 87159 genes based on the codes and supplementary information provided in the ZUPLS study [5]. For *leave-one-species-out* validation, we used the same set of features and considered only the 87159 genes belonging to 27 species that had available network information.

### Classification and evaluation

For classification, we used Support Vector Machine (SVM) [29] with a radial basis function kernel, and a grid search was done to find the best parameters. All our codes were written in Python and used the SVM implementation from the scikit-learn Python package [28]. For *leave-one-species-out* validation, we used RFC implementation from scikit-learn Python package [28] since it was easily scalable across 27 organisms.

Essential genes are in general present in lower fraction compared to non-essential genes (as can also be seen from S1 Table). In order to account for this class imbalance in the dataset, it is important to undersample non-essential genes (or oversample essential genes) for effective evaluation of any classification method. Random undersampling, synthetic minority over-sampling technique (SMOTE) and Adaptive synthetic sampling approach (ADASYN) are the different sampling strategies that have been previously used for the task of essential gene prediction [6, 7]. We followed the random undersampling strategy followed by Liu *et al*. [6] to enable a performance comparison of our network-based features.

Given the class imbalance in the dataset, accuracy is not a good measure to assess the performance of a classifier. In a class-imbalanced binary classification problem, a higher accuracy value is possible even if the classifier labels all items as belonging to the majority class. In order to tackle this problem, we used better metrics such as AUROC (Area under the Receiver-Operator Characteristic Curve), precision and recall, considering essential genes as the positive class.

1. **Area Under the curve of the Receiver Operating Characteristic (AUROC)** measures the area under a plot of False Positive Rate (FPR) versus True Positive Rate (TPR), for the same classification problem, at different classification thresholds. The best AUROC curve will have low False Positive Rate (0) and a high True Positive Rate (1) and hence an AUROC of 1.0. The AUROC quantifies the performance of the model. The more the AUROC, the better the performance of the model.
2. **Precision** quantifies the number of true positives among the predicted positives.
3. **Recall** quantifies the number of true positives out of the total number of positive class elements in the data.
4. **Area Under the Precision Recall Curve (AUPRC)** measures the area under precision vs recall curve at different classification thresholds.

We evaluated our method by comparing AUROC, Precision, Recall and AUPRC measures with the baseline methods. We performed statistical comparisons by means of a one-tailed *Z*-test, to evaluate the significance of different metrics. This tests the mean of the 50 values obtained during 50 undersamplings of one method versus another.

We compared our approaches with Liu *et al*. [6], ZUPLS [5] and the conventional network feature set (“naïve network baseline”) used in previous studies [11, 12]. These network features are degree centrality, closeness centrality, clustering coefficient and betweenness centrality. We focus only on these baselines since we are not using any expression data or function related information. We also didn’t use many centrality measures proposed for the purpose of ranking based approaches since they were created using either biological domain information or expression-related information and usually ranked genes within an organism. We set out to verify features that are effective across a diverse set of organisms using plain sequence and network information.

## Results

In this section, we establish that our network-based features are highly informative and enable better classification of essential proteins compared to all previous methods. We further show that the addition of sequence-based features is able to further improve performance. We finally propose that the simplified features obtained using LASSO is an effective feature set for performing predictions of essentiality in newer organisms. Our results are discussed in detail in the following sections.

### Classification using ReFeX features

While generating ReFeX features, the algorithm terminated at a different number of iterations for networks of different organisms and consequently, yielded a different set of recursive features. We took the organism that had the smallest number of recursive features (36) and generated the 36 features for all the other organisms. This gave an AUROC of 0.578, a precision of 0.162 and recall of 0.338. We also took the organism with the maximum number of recursive features (93) and generated the 93 features for all the other organisms. This gave better results (AUROC: 0.817, Precision: 0.322, Recall: 0.718). When we combined all the unique recursive features across all the 27 organisms (267 features), the performance improved further (AUROC: 0.838, Precision: 0.321, Recall: 0.754). We conclude that a diverse set of ReFeX features, thus generated, are effective in predicting essential genes across organisms.

### Classification using network centrality measures

We also performed classification using a combined set of “12 centrality measures”, as described in Methods. Classification using this feature set resulted in an AUROC of 0.830 and a precision of 0.317 as can be seen from Table 1. Further, we find that most of the centrality measures are significantly higher for essential proteins. To evaluate the significance of centrality measures, we compared the mean of each measure for essential genes with randomly (sub-)sampled non-essential genes and computed a *p*-value. This bootstrapped *p*-value is computed as the fraction of iterations in which the mean centrality measure for a random sub-sample of non-essential genes is greater than or equal to the mean centrality measure of essential genes.

**Table 1:** Performance comparison of various feature sets for classification of essential genes.

Method/Feature set	AUROC	Precision	Recall
**Liu *et al*.**	0.784	0.254	0.688
**ZUPLS**	0.705	0.255	0.663
**Naïve network baseline**	0.800	0.289	0.702
**ReFeX 267 features (ReFeX feature set)**	0.838	0.321	0.754
**12 centrality measures**	0.830	0.317	0.733
**14 network measures**	0.835	0.321	0.742
**283 network properties**	**0.847**	**0.320**	**0.773**
**100 selected network properties**	**0.844**	**0.316**	**0.775**
**597 sequence and network properties**	**0.857**	**0.335**	**0.769**
**300 selected sequence and network properties**	**0.857**	**0.332**	**0.771**
**Top 200 selected sequence and network properties**	**0.860**	**0.334**	**0.779**
**Top 100 selected sequence and network properties**	**0.859**	**0.337**	**0.771**

The values in bold highlight the better-performing methods, based on AUROC, Precision and Recall measures. This table summarises the results of the different network-based features with Liu *et al*., ZUPLS and naïve network baselines. We can see that combined network properties, “12 centrality measures”, “14 network measures” and “ReFeX feature set” are effective in transferring essential genes across organisms, as compared to all the baseline methods. We can also see that adding sequence-based to network-based features yields more improvement in performance. Note that all the improvements over the baseline are statistically significant, as we show in S5 Table as described in Methods. The higher set of features included a smaller subset of features and are significantly better as shown in S6 Table. Area Under the curve of the Receiver Operating Characteristic (AUROC) measures the area under the plot of False Positive Rate vs True Positive Rate, Precision = True Positive/ (True Positive+False Positive), Recall = True Positive/ (True Positive+False Negative), Area Under Precision Recall Curve (AUPRC) measures the area under the plot of precision vs recall curve and the results are in S7 Table.

Through this process, we identified significant centrality measures in each network. We also conducted the Wilcoxon Rank-Sum test [30] to test for the significance of the centrality measures. In this way, we evaluated significant centrality properties across 27 networks and found commonly significant centrality measures across all the networks. Table 2 lists all the measures used in our set of “12 centrality measures” and the number of organisms in which they were found to be significant. These centrality measures are found to be associated with lethality based on our study across 27 organisms and reaffirm our earlier observations on the “centrality–lethality” hypothesis that it holds true for a large number of organisms [23].

**Table 2:** Centrality measures and their significance across 27 organisms.

Centrality measure	Bootstrap test	Wilcoxon Rank-Sum test
Edge Clustering Coefficient Centrality	0	8
Betweenness Centrality	27	23
Load Centrality	27	24
Random Walk Betweenness Centrality	19	25
Information Centrality	19	26
Closeness Centrality	27	26
Degree Centrality	27	26
Harmonic Centrality	27	26
PageRank	27	26
Reaching Centrality	27	26
Subgraph Centrality	27	26
Eigenvector Centrality	27	27

Table shows the number of organisms in which a given measure was found to be significant (*p*-value <0.05). For further details on *p*-value computation, refer text.

### Complete set of network properties improves performance

The set of “14 network measures” gave an AUROC of 0.835 and a precision of 0.321. These approaches and “12 centrality measures” set discussed previously are highly scalable approaches to predict essential genes with a few sets of network properties. When we combined all the 283 network properties, we achieved the best performance with AUROC of 0.847 and precision of 0.320. This shows that a diverse set of network properties that capture the global, local and neighbourhood information can effectively predict essential genes across different networks. ReFeX captures local and neighbourhood information recursively. Centrality measures capture global information. Local properties such as weighted degree and clustering coefficient capture the local properties of a node. A list of all 283 network properties along with their LASSO coefficients is available in S3 Table.

### Augmenting network with sequence features further improves performance

Through the above studies, we find that network properties outperform sequence-based properties. To examine whether combining sequence-based features with network-based improves performance, we combined sequence and network properties for predicting essential genes. This gave better AUROC (0.857), precision (0.335) and recall (0.769) values than combined network-based features.

### Reduced set of features using LASSO achieve comparable performance

It is also clear from Table 1 that 100 selected network features using LASSO gave comparable performance to the entire set of combined 283 network features. Also, the selected 300 combined network and sequence properties using LASSO are equally effective as the entire set of 597 properties. LASSO drives weights of 297 out of 597 features to zero; this is perhaps because the features do not contain any useful information or contain only information already captured in the selected 300. Thus, the top features that are selected for classification perform nearly as well as the entire set of 597. A list of all 597 network properties along with their LASSO coefficients is available in S4 Table.

### Top ranked set of features using LASSO are equally effective

We also tried ranking the features using LASSO coefficients and selected the top ones. We found that the top 200 selected sequence and network features gave similar performance to the 300 LASSO selected network and sequence features with non-zero weights. We also found that the top 100 selected sequence and network properties are equally effective as the 300 LASSO selected network and sequence properties with non-zero weights. The performance did not deteriorate, suggesting that the top features are sufficient to perform better classification.

### Classification using *leave-one-species-out* validation

We also tried *leave-one-species-out* validation in which we found network-based features to be better than the sequence-based baselines. The results are in S8 Table. We can clearly see from the results that the proposed set of features are significantly effective across all 27 organisms over the baseline methods. We can also find that the combined sequence and network properties are quite effective in a majority of the organisms.

## Discussion

The prediction of essential genes in an organism is a challenging machine learning problem. Many previous studies have tackled this problem by engineering various types of features, from sequence to network [3, 4]. However, the network-based features employed in previous studies are somewhat simplistic and do not tend to capture the complexities of PPI structure. Therefore, in this study, we set out to investigate various network features for their ability to enable discrimination of essential and non-essential genes across several organisms. Using essentiality data from DEG and PPI data from the STRING, we outline several interesting network-based features that are able to greatly enhance classification performance. Overall, our approach statistically significantly outperforms the best reported results at matching the DEG as a gold standard, by using features derived from local, neighbourhood and global network properties, and is also useful for predicting essential genes across organisms.

Our key results are three-fold. First, we show that network-based properties are able to predict essential genes across organisms better. Notably, they outperform sequence-based features by a distance. Additionally, we show that a LASSO-based feature selection that yields a reduced set of top features is able to perform better as well. Second, we show that even a few network properties, such as those given by “12 centrality measures”, are able to aid greatly in classification. Finally, we show that augmenting the network-based features with sequence features further improves classification at the cost of an increased number of features, and is effective across organisms. Importantly, obtaining sequence-based orthology features requires pairwise comparison of genomes that is computationally expensive. Also, our reduced top ranked set of 100 sequence and network features could be highly useful to predict essential genes in a new organism.

Across-organism methods are particularly interesting since they help in utilising prior information from all the available essentiality studies conducted on different organisms; extract the universal set of features and transfer it to new organisms. Essential genes are effectively transferred in closely-related organisms since they share a lot of orthologous genes. However, the number of organisms with experimental data on essential genes is very sparse. Hence, this approach cannot be applied on a large scale. Also, essential genes are transferred across distantly-related organisms [10]. However, these approaches are effective in few pairwise transfers, but they need not generalise for all pairs of organisms since orthology accounts for an only small portion of the genome. In addition, genes show variations in gene regulations and functions across distantly-related organisms [4]. Scaled network-based features are potentially robust to these factors and are hence highly effective in predicting essential genes across organisms.

STRING networks are obtained from genomic channels which inherently have some sequence information. However, adding sequence-based features yields further improvement suggesting that some residual information could be missing in these genomic channels that are explicitly captured in sequence-based features. Also, hub proteins are found to evolve slowly and are mostly essential [31]. While the evolutionary information of an organism can be obtained from the co-occurrence network that is one of the evidence channels used in constructing STRING networks, the role played by the protein is characterised by the underlying network-features that accounts for both the network position as well as the evolutionary context of that protein. So, these protein interaction networks offer a much better perspective incorporating the evolutionary and genomic information as well.

As with any approach to computationally predict gene essentiality, our study also has its limitations. Firstly, we are limited by the quality of STRING PPI data. The PPI data is obtained from various evidence channels, yet they are prone to false positives and data incompleteness. The other bias could be that well-studied genes might have more interaction partners than poorly studied genes. The conclusions are based on balanced undersampled datasets across 27 diverse organisms. Lastly, our conclusions need not be universal since we studied only 27 bacterial species based on the available essentiality data. However, as more experimental data on essentiality become available, it will be possible to further test our approach.

It is also important to note that the experimental identification of essential genes itself remains a work in progress, and there remain major variations between multiple studies reporting essential genes on similar media, for identical strains. Our notion of gene essentiality essentially pertains to the consolidated experimental data available via DEG 11.1. However, as better and more reliable data accumulate from newer experiments, it is likely that we will be able to build better models and consequently, predict essential genes with higher accuracies.

Nevertheless, our proposed set of features can be derived for any organism containing both sequence and interactome information such as those in STRING [14]. The extracted features can be used to predict essential genes in any organism lacking experimental information on essential genes.

## Conclusion

The central contribution of this study is the engineering of several potent network-based features for predicting gene essentiality across organisms. Notably, we have adapted algorithms such as ReFeX to better predict gene essentiality based on local, global and neighbourhood properties. Further, we find very small feature sets, such as the “12 centrality measures” and “14 network measures”, which provide excellent discriminative power. Adding sequence-based features to network-based features yields a further improvement and our selected set of 100 network and sequence features could be the most useful set for predicting essential genes in newer organisms. We also reported a *leave-one-species-out* validation, which demonstrates the proposed sets of features to be effective for performing predictions across organisms. Notably, network-based features can probably point us towards uncovering the key roles played by the essential nodes in network structure.

## Supporting information

S1 Dataset Supplementary data. PPI Data of 27 organisms along with essential genes information.
(ZIP)

S1 Table Organism statistics. List of 27 organisms along with their statistics.
(XLSX)

S2 Table ReFeX features. List of 267 ReFeX features.
(XLSX)

S3 Table 283 network properties. List of 283 network features sorted according to their LASSO coefficients.
(XLSX)

S4 Table 597 network and sequence properties. List of 597 network and sequence features sorted according to their LASSO coefficients.
(XLSX)

S5 Table Statistical tests comparing AUROC, precision and recall for different methods with baselines, along with their mean, standard deviation, *Z*-score and *p*-values. (XLSX)

S6 Table Statistical tests comparing AUROC, precision and recall within our methods, along with their mean, standard deviation, *Z*-score and *p*-values. (XLSX)

S7 Table AUPRC results of various methods. Table containing AUPRC of undersampling evaluation results.
(XLSX)

S8 Table *Leave-one-species-out* results. Table containing AUROC of *leave-one-species-out* results.
(XLSX)

## References

[1] Rancati G, Moffat J, Typas A, Pavelka N. Emerging and evolving concepts in gene essentiality. Nat Rev Genet. 2017;19:34–49. 10.1038/nrg.2017.74 29033457 10.1038/nrg.2017.74

[2] Juhas M, Eberl L, Glass JI. Essence of life: essential genes of minimal genomes. Trends Cell Biol. 2011;21(10):562–568. 10.1016/j.tcb.2011.07.005. 21889892

[3] Zhang X, Acencio ML, Lemke N. Predicting Essential Genes and Proteins Based on Machine Learning and Network Topological Features: A Comprehensive Review. Front Physiol. 2016;7:75 10.3389/fphys.2016.00075 27014079 10.3389/fphys.2016.00075 PMC4781880

[4] Mobegi FM, Zomer A, de Jonge MI, van Hijum SAFT. Advances and perspectives in computational prediction of microbial gene essentiality. Brief Funct Genomics. 2017;16(2):70–79. 10.1093/bfgp/elv063 26857942 10.1093/bfgp/elv063

[5] Song K, Tong T, Wu F. Predicting essential genes in prokaryotic genomes using a linear method: ZUPLS. Integr Biol. 2014;6:460–469. 10.1039/C3IB40241J 10.1039/c3ib40241j 24603751

[6] Liu X, Wang BJ, Xu L, Tang HL, Xu GQ. Selection of key sequence-based features for prediction of essential genes in 31 diverse bacterial species. PLoS ONE. 2017;12(3):e0174638 10.1371/journal.pone.0174638 28358836 10.1371/journal.pone.0174638 PMC5373589

[7] Yu Y, Yang L, Liu Z, Zhu C. Gene essentiality prediction based on fractal features and machine learning. Mol BioSyst. 2017;13:577–584. 10.1039/c6mb00806b 28145541 10.1039/c6mb00806b

[8] Nigatu D, Sobetzko P, Yousef M, Henkel W. Sequence-based information-theoretic features for gene essentiality prediction. BMC Bioinformatics. 2017;18(1):473 10.1186/s12859-017-1884-5 29121868 10.1186/s12859-017-1884-5 PMC5679510

[9] Hwang YC, Lin CC, Chang JY, Mori H, Juan HF, Huang HC. Predicting essential genes based on network and sequence analysis. Mol BioSyst. 2009;5:1672–1678. 10.1039/B900611G 19452048 10.1039/B900611G

[10] Deng J, Deng L, Su S, Zhang M, Lin X, Wei L, et al Investigating the predictability of essential genes across distantly related organisms using an integrative approach. Nucleic Acids Res. 2011;39(3):795–807. 10.1093/nar/gkq784 20870748 10.1093/nar/gkq784 PMC3035443

[11] Cheng J, Xu Z, Wu W, Zhao L, Li X, Liu Y, et al Training set selection for the prediction of essential genes. PLoS ONE. 2014;9(1):e86805 10.1371/journal.pone.0086805 24466248 10.1371/journal.pone.0086805 PMC3899339

[12] Cheng J, Wu W, Zhang Y, Li X, Jiang X, Wei G, et al A new computational strategy for predicting essential genes. BMC Genomics. 2013;14(1):910 10.1186/1471-2164-14-910 24359534 10.1186/1471-2164-14-910 PMC3880044

[13] Lin Y, Zhang RR. Putative essential and core-essential genes in Mycoplasma genomes. Sci Rep. 2011;1:53 10.1038/srep00053 22355572 10.1038/srep00053 PMC3216540

[14] Szklarczyk D, Franceschini A, Wyder S, Forslund K, Heller D, Huerta-Cepas J, et al STRING v10: protein–protein interaction networks, integrated over the tree of life. Nucleic Acids Res. 2014;43(D1):D447–D452. 10.1093/nar/gku1003 25352553 10.1093/nar/gku1003 PMC4383874

[15] Luo H, Lin Y, Gao F, Zhang CT, Zhang R. DEG 10, an update of the database of essential genes that includes both protein-coding genes and noncoding genomic elements. Nucleic Acids Res. 2014;42(D1):D574–D580. 10.1093/nar/gkt1131 24243843 10.1093/nar/gkt1131 PMC3965060

[16] Chen WH, Lu G, Chen X, Zhao XM, Bork P. OGEE v2: an update of the online gene essentiality database with special focus on differentially essential genes in human cancer cell lines. Nucleic Acids Res. 2017;45(D1):D940–D944. 10.1093/nar/gkw1013 27799467 10.1093/nar/gkw1013 PMC5210522

[17] Henderson K, Gallagher B, Li L, Akoglu L, Eliassi-Rad T, Tong H, et al. It’s who you know: Graph mining using recursive structural features In: Proceedings of the ACM SIGKDD International Conference on Knowledge Discovery and Data Mining; 2011. p. 663–671.

[18] Henderson K, Gallagher B, Eliassi-Rad T, Tong H, Basu S, Akoglu L, et al. Rolx: structural role extraction & mining in large graphs. In: Proceedings of the 18th ACM SIGKDD international conference on Knowledge discovery and data mining. ACM; 2012. p. 1231–1239.

[19] Gupte PV, Ravindran B, Parthasarathy S. Role Discovery in Graphs Using Global Features: Algorithms, Applications and a Novel Evaluation Strategy. In: 2017 IEEE 33rd International Conference on Data Engineering (ICDE); 2017. p. 771–782.

[20] Franceschini A, Szklarczyk D, Frankild S, Kuhn M, Simonovic M, Roth A, et al STRING v9. 1: protein-protein interaction networks, with increased coverage and integration. Nucleic Acids Res. 2012;41(D1):D808–D815. 10.1093/nar/gks1094 23203871 10.1093/nar/gks1094 PMC3531103

[21] Jeong H, Mason SP, Barabási AL, Oltvai ZN. Lethality and centrality in protein networks. Nature. 2001;411(6833):41 10.1038/35075138 11333967 10.1038/35075138

[22] Ning K, Ng HK, Srihari S, Leong HW, Nesvizhskii AI. Examination of the relationship between essential genes in PPI network and hub proteins in reverse nearest neighbor topology. BMC Bioinformatics. 2010;11(1):505 10.1186/1471-2105-11-505 20939873 10.1186/1471-2105-11-505 PMC3098085

[23] Raman K, Damaraju N, Joshi GK. The organisational structure of protein networks: revisiting the centrality–lethality hypothesis. Syst Synth Biol. 2014;8(1):73–81. 24592293 10.1007/s11693-013-9123-5 PMC3933631

[24] Barabási AL, Pósfai M. Network science. Cambridge: Cambridge University Press; 2016. Available from: http://barabasi.com/networksciencebook/.

[25] Wang J, Li M, Wang H, Pan Y. Identification of Essential Proteins Based on Edge Clustering Coefficient. IEEE/ACM Transactions on Computational Biology and Bioinformatics. 2012;9(4):1070–1080. 10.1109/TCBB.2011.147 22084147 10.1109/TCBB.2011.147

[26] Tibshirani R. Regression Shrinkage and Selection via the Lasso. J R Stat Soc Series B (Methodological). 1996;58(1):267–288. 10.1111/j.2517-6161.1996.tb02080.x

[27] Breiman L. Random Forests. Mach Learn. 2001;45(1):5–32. 10.1023/A:1010933404324

[28] Pedregosa F, Varoquaux G, Gramfort A, Michel V, Thirion B, Grisel O, et al Scikit-learn: Machine Learning in Python. J Mach Learn Res. 2011;12:2825–2830.

[29] Cortes C, Vapnik V. Support-vector networks. Mach Learn. 1995;20(3):273–297. 10.1007/BF00994018

[30] Mann HB, Whitney DR. On a Test of Whether one of Two Random Variables is Stochastically Larger than the Other. Ann Math Statist. 1947;18(1):50–60. 10.1214/aoms/1177730491

[31] Hahn MW, Kern AD. Comparative genomics of centrality and essentiality in three eukaryotic protein-interaction networks. Mol Biol Evol. 2004;22(4):803–806. 10.1093/molbev/msi072 15616139 10.1093/molbev/msi072

